# Total phenolic, flavonoid content, and antioxidant activity of bulbs, leaves, and flowers made from *Eleutherine bulbosa* (Mill.) Urb

**DOI:** 10.1002/fsn3.834

**Published:** 2018-12-21

**Authors:** Peiqi Shi, Wenjun Du, Yuanyuan Wang, Xingxing Teng, Xiaodong Chen, Lianbao Ye

**Affiliations:** ^1^ School of Pharmacy Guangzhou key laboratory of construction and application of new drug screening model systems Guangdong Pharmaceutical University Guangzhou Guangdong China; ^2^ School of Pharmacy Jiangxi University of Traditional Chinese Medicine Nanchang Jiangxi China

**Keywords:** antioxidant activity, *E. bulbosa*, total flavonoids, total phenols

## Abstract

In the current investigation, total phenols and flavonoids contents of *Eleutherine bulbosa* (Mill.) Urb. bulbs, leaves, and flowers were quantified by Folin–Ciocalteu's and borohydride/chloroquinone methods, respectively. Antioxidant activity of the plant extracts was evaluated by means of peroxide scavenging capacity assay and by cell antioxidation method. Antioxidant activity of *E. bulbosa* bulbs, leaves, and flowers was correlated with total phenols and flavonoids. The total phenols and flavonoids of the bulbs of *E. bulbosa* were higher than leaves and flower and its antioxidant activity was also stronger than leaves and flowers of *E. bulbosa*. The higher content of flavonoids or total phenols, the stronger the antioxidant capacity in vitro. The antioxidant activity of *E. bulbosa* extract showed it's certain nutritional value and therefore had the potential as a source of natural antioxidants.

## 1 INTRODUCTION

*Eleutherine(E.) bulbosa* (Mill.) Urb belongs to the Iredaceae *Eleutherine*. *Eleutherine bulbosa* was also reported in the literature as *Eleutherine plicata* (Goldblatt & Snow, 1991). The research suggested that the plant of Iredaceae *Elertherine* included four species, namely *E. bulbosa*,* E. latifolia*,* E. angusta*, and *E. citriodora* (Xu, He, Zeng, Zhang, & Wang, 2012). *Eleutherine bulbosa* was widely cultivated throughout the world especially in Africa and Asia for its medicinal properties (Rani & Nair, 2016). Some researchers pointed out that *Eleutherine americana*,* E. plicata*, and *E. bulbosa* were the same species (Guerra, 1988 and Insanu, Kusmardiyani, & Hartati, 2014). In China and South‐East Asia, the bulbs of *E. bulbosa* were widely used as medicine and played an important role in medical care and disease treatment. It could be used for treating hemoptysis, dysentery, irregular menstruation, abdominal pain, rheumatalgia, traumatic injury, and sore boil (Min, Wu, & Zhu, 2015). *Eleutherine bulbosa* is an Iridaceae popularly known in the Amazonian region where it exists in the form of a clump with red bulbs like an onion and its leaves are entire, pleated, and simple, and its flowers are colored in white to pink and the red bulbs are widely used in Brazilian folk phototherapy, especially in the Amazonian region (Malheiros, Mello, & Barbosa, 2015). *Eleutherine bulbosa* is native to South America and is present in tropical countries. The bulbs were used as an abortifacient, emmenagogue, a purgative, and an anticancer drug in folk medicine (Padhi & Panda, 2015). The *E. bulbosa* extracts had a variety of biological activities including antibacterial, antifungal, anti‐amoebaean, antioxidation, anti‐inflammatory, and analgesic (Do et al., 2014 and Pan et al., 2011). It was reported that the bulbs of *Eleutherine* (*E. bulbosa* and *E. americana*) contained naphtoquinones such as elecanacine, eleutherine, eleutherol, and eleutherinone, which were usually existed in cell vacuoles in the form of glycosides and had antimicrobial, antifungal, antiviral, anticancer, antioxidant, and antiparasitic properties (Dai, Min, Zhong, & Wang, 2013). The main components of the *E. bulbosa* were naphthalene, naphthol, and anthraquinone (Betteridge, 2000 and Wang, Wang, He, & Wang,^2015^). The oxidative stress of human body played an important role in the health. In normal conditions, the level of oxygen reduction in the human body meant that the generation of free radicals was in equilibrium with the antioxidant system and did not produce oxidative stress (Laghabenamrouche & Madani, 2013). Due to the oxidative stress, many biological molecules such as fatty acids, proteins, nucleic acids, and carbohydrates were impaired which led to cellular damage and a variety of physiological and pathological abnormalities. For example, the development of aging, neurological diseases, cardiovascular diseases, inflammation, and other diseases was highly related to oxidative stress. Elimination of oxidative stress became an important research direction in the prevention and treatment of related diseases (Deighton, Brennan, Finn, & Davies, 2000; Pashkow, 2011 and Zhao & Zhao, 2013). The widespread presence of secondary metabolites, antioxidant phenols and flavonoids in many fruits, vegetables, and herbs had been proven to be effective in counteracting oxidative stress. Phenols and flavonoids could prevent oxidative degradation of fatty acids (Hossain & Shah, 2015 and Ifesan, Siripongvutikorn, & Voravuthikunchai, 2009). Due to the increasing demands for natural antioxidants and food preservatives, the antioxidant properties of medicinal plants attracted more and more attention (Bhadauria & Kumar, 2011). We inferred that the therapeutic effect of the disease on *E. bulbosa* could be related to its antioxidant activity. In the current investigation, total phenols and flavonoids contents of *E. bulbosa* (Mill.) Urb. bulbs, leaves, and flowers were quantified by Folin–Ciocalteu's and borohydride/chloroquinone methods, respectively. Antioxidant activity of extracts was evaluated by means of peroxide scavenging capacity (PSC) assay and by the cell antioxidation method.

## 2 MATERIALS AND METHODS

### 2.1 Raw materials

Raw material of *E. bulbosa* was provided by Guangzhou red lily Co., Ltd. Bulbs and leaves were collected in November every year. After harvested, it was dried in an oven at 60°C.

### 2.2 Standards and reagents

Folin reagent, ascorbic acid, 2,7‐dichlorofluorescein dimerquinone(DCFH‐DA), fluoresce in disodium salt, sodium borohydride (reagent grade), tetrachlorobenzene quinone, vanillin, quercetin, catechins, acetocaustin, and methyl alcohol were purchased from Sigma‐Aldrich. Gallic acid (analytical purity) was purchased from ICN biological medicine. 2, 2′‐azobisisobutyramidine dihydrochloride (ABAP) was supplied by Wako Public Works. Other solvents and reagents were purchased from commercial vendors and were dried and purified by conventional methods prior to use. HepG2 cells were purchased from the United States Institute of Culture (ATCC). Hanks’ broth was supplied by Gibco Biotech's product. Fetal bovine serum was obtained from Atlanta Biotechnology Company.

### 2.3 Extraction of free phenols

About 2 g raw material was weighed and transferred to 50 ml of ice‐cold 80% aqueous acetone (50 ml) was added, and then the mixture was homogenized for 10 min and centrifuged at 3,500 *g* to collect the liquid supernatant. The pellet was further extracted with ice‐cold 80% aqueous acetone (50 ml). The two supernatants were combined and evaporated at 45°C with a rotary evaporator. The residue was diluted to 10 ml with deionized water and restored at −20°C.

### 2.4 Extraction of combined phenols

The 2 ml of 2 mol/L sodium hydroxide solution was added to precipitation, and the mixture was blown for 2 min with nitrogen flow, stirred for 1 hr, followed by addition of 4 ml of concentrated hydrochloric acid and mixed for a few minutes. The 20 ml n‐hexane was added, and the mixture was shaken for 10 min, centrifuged at 3500 rpm for 5 min. The liquid supernatant was removed. The mixture was extracted with ethyl acetate (20 ml ×5). The organic layer was condensed. The deposit was diluted to 10 ml with deionized water and restored at −20°C.

### 2.5 Extraction of free flavonoids

About 2 g sample powders were accurately weighed with an analytical balance, transferred to 50 ml centrifuge tube and followed by addition of 70% ethanol. The mixture was refluxed for 1 hr and extracted subsequently with petroleum ether, ether, and ethyl acetate. Petroleum ether fraction was discarded, and ether fraction was diluted to required concentration.

### 2.6 Extraction of bound flavonoids

Ethyl acetate fraction of the samples was hydrolyzed with 7% H_2_SO_4_ for 2 hr and was re‐extracted with fresh ethyl acetate. The sample was diluted to required concentration.

### 2.7 Determination of total phenols

The total phenols content of the extract was determined by the Folin–Ciocalteu's method. The concentration gradient of gallic acid was prepared as standard solution (0–200 g/ml), and calibration curve was established using gallic acid. The free phenol samples of bulbs, leaves, and flowers were diluted with deionized water as sample solutions, in which bulbs and leaves samples were diluted to 20‐fold and flowers were diluted to 80‐fold. The 400 μl of deionized water and 100 μl of sample or standard solution was added to 5‐ml centrifuge tube and mixed well. The diluted extract or gallic acid was added to 0.2 ml Folin–Ciocalteu reagent and mixed for 6 min, followed by the addition of 2 ml of 20% sodium carbonate and 800 μl deionized water. The mixture was placed for 90 min at room temperature. Every experiment was performed in triplicate. The absorbance of the mixture was measured at 760 nm using a UV spectrophotometer. The concentration of free phenols and bound phenols in the sample was calculated, according to the regression equation of standard curve. The sum concentration of free phenols and bound phenols was total phenols content, and the results were expressed as mg of gallic acid equivalent per 100 g dry weight.

### 2.8 Determination of total flavonoids

The content of total flavonoids was investigated using the sodium borohydride/chloroquinone method. Catechin standard solution (0–6 mM) was prepared with tetrahydrofuran/ethanol (1:1, v/v). The 2 ml sample was taken in 10‐ml glass tubes and dried by fluxing nitrogen. Then, it was dissolved in 3 ml tetrahydrofuran/ethanol (1:1, v/v). The mixture of 1 ml this solution, 0.5 ml sodium borohydride(50 mM), and 0.5 ml aluminum trichloride (74.6 mM) were shaken for 15 min at room temperature. Then, 0.5 ml sodium borohydride was further added to each test tube and shaken for 30 min at room temperature. Cold acetic acid solution was added into each tube, and the solution was kept in the dark for 15 min. Then, 1 ml of 20 mM chloranil was added and heated at 95°C. The reaction solutions were cooled, and the final volume was diluted to 4 ml with methanol. Then, 1 ml of 16% vanillin methanol solution was added into each tube, followed by addition of 2 ml hydrochloric (12 mM), and the reaction solutions were kept in the dark for 15 min after a thorough mix. Finally, the mixture was centrifuged at 3500 rpm for 3 min and the absorbance was measured at 490 nm by spectrophotometer. Each concentration level of standard solutions or sample was repeated for three times to obtain the average value. The concentration of flavonoids in the sample was calculated according to the regression equation of the standard curve. The sums of free and bound flavonoids were total flavonoids content, and the results were expressed.

### 2.9 Antioxidant capacity

#### 2.9.1 Peroxide scavenging capacity assay

Antioxidant capacity was determined by PSC assay. Standard solutions (1–6.3 μg/ml) of ascorbic acid were prepared and diluted to the experimental concentration by buffer solution. Five concentration gradient of sample solution was prepared according to standard solution. A solution of 400 μl DCFH‐DA (25.625 mM) in 3.74 ml methanol was divided and stored at −40°C. The mixture of 893 μl potassium hydrate (1.0 mM) and 107 μl DCFH‐DA (2.48 mM) was added to 10‐ml test tube, and then 7 ml buffer solution was added to the mixture to obtain DCFH solution. About 100 μl samples or standard solutions, 100 μl DCFH solution, and 50 μl 200 mM ABAP solution were added to each well of 96‐well plate, respectively. Standard or sample solution immediately was placed on a fluorescent microplate reader. Read the release light of 538 nm every 5 min with the excitation light at 485 nm and read no less than 12 times. Antioxidant capacity was expressed as mg of ascorbic acid equivalent per 100 g dry weight.

#### 2.9.2 Antioxidant capacity in cell

Cell antioxidant assay capacity. Antioxidant capacity was determined by cell antioxidant capacity assay. HepG2 cells were cultured in CO_2_ incubator with RPMI‐1640 medium that contains 10% fetal bovine serum and was maintained at 37°C. HepG2 cells were seeded at a density of 6 × 10^4^ per well on 96‐well plates in 100 μl of growth medium. The outside wells of the plate were not used since there was much more variation from them than from the inner well. After 24 hr seeding, the growth medium was removed and the wells were washed with PBS. The requirement concentration (0.025–0.5 mg/ml) of propolis alcohol was obtained by fresh RPMI‐1640 culture medium, propolis alcohol solution, and DCFH‐DA fluorescent probe solution and each mass concentration had five wells. The final concentration of DCFH‐DA was 25 mol/L and was cultured for 1 hr in 5% CO_2_, 37°C incubator. The cells were cleaned with 100 μl PBS in triplicate, and the final concentration of ABAP was 600 μM by adding ABAP working fluid. The 96‐well plate was placed into a fluorescent microplate reader. Emission at 538 nm was measured with excitation at 485 nm every 5 min for 1 hr. The control group was treated with DCFH‐DA and ABAP, without propolis alcohol extract. The blank group was treated with DCFH‐DA, but without adding ABAP and propolis alcohol extract.

### 2.10 Statistical analyses

Sigma plot 12.5 and CalcuSyn 2.0 and other analysis software were used to process the data to calculate the PSC value. The data of the experiment were reported as the mean ± *SD* for at least three replicates for each sample. Differences were considered to be significant when *p* < 0.05. Origin 8.0r and other analysis software were used to process the data. The area of the time‐fluorescence intensity curve was calculated using Origin 8.0 software and the intracellular antioxidant activity (CAA) was calculated according to follow equation:

CAA unit=1−∫SA/∫SC

where ∫SA is the integrated area under fluorescence versus time curve from the propolis alcohol extract with different concentrations were added and ∫SC is the integral area of the fluorescence versus time curve from control group. All tests were performed in triplicate. The CAA value is obtained by converting EC_50_. In each experiment, quercetin was used as a standard, and cellular antioxidant activities were expressed as quercetin equivalents per 100 μmol of dry weight.

## 3 RESULTS AND DISCUSSION

### 3.1 Total phenol content

**Figure 1:**
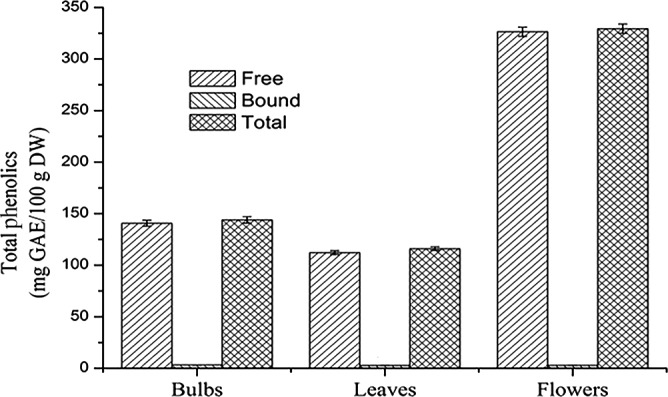
Comparison of free phenol, bound phenol, and total phenols

The total phenol contents of *E. bulbosa* were quantified. The regression equation of standard curve of gallic acid was *y* = 0.0045*x *+ 0.0051 with *R* ^2^ = 0.9991. The results showed that the linear relationship was good in the detection ranges. *Eleutherine bulbosa* flowers had the highest free phenols content (326.55 ± 4.48 mg GAE/100 g DW), total phenols content (329.45 ± 4.55 mg GAE/100 g DW), followed by *E. bulbosa* bulbs and leaves. The total phenol contents of flowers was almost three times compared with the content of bulbs and leaves. The bound phenols (3.23 ± 0.15 mg GAE/100 g DW) were found to be highest in *E. bulbosa* bulbs followed by flowers and leaves. As shown in Figure 1, phenols of *E. bulbosa* were mainly in free state, bound phenols were almost negligible. The results indicated that the flowers were more antioxidants than bulbs and leaves.

### 3.2 Total flavonoids content

**Figure 2:**
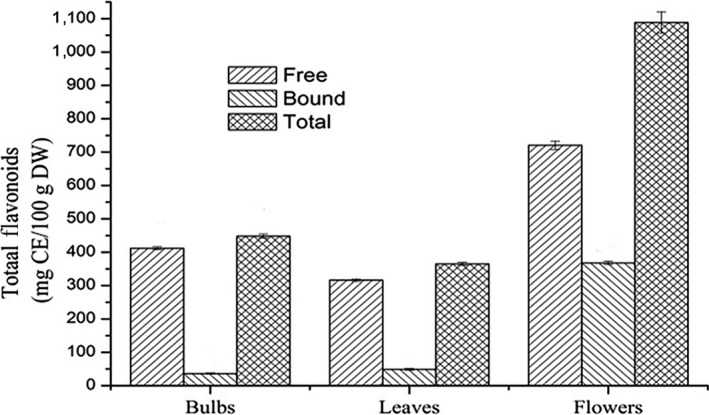
Comparison of free flavonoids, bound flavonoids, and total flavonoids

The total flavonoid contents of *E. bulbosa* were quantified. The regression equation of standard curve of catechin was *y* = 0.1208*x *+ 0.0058 with *R* ^2^ = 0.9966. The results showed that the linear relationship was good in the detection range. The total flavonoid contents of *E. bulbosa* were as followed: leaves (365.20 ± 4.58 mg CE/100 g DW) < bulbs (448.24 ± 5.89 mg CE/100 g DW) <flowers (1088.33 ± 32.64). The content of total flavonoids in *E. bulbosa* was the highest in flowers (Figure 2), which was 2.5 times in bulbs and three times in leaves. The content of flavonoids in bulbs and leaves was mainly free state and the bound flavonoids nearly could be negligible. However, the content of flavonoids in flowers was 33.8%. Flavonoids have important physiological value to human health.

### 3.3 Determination of antioxidant capacity of *E. plicata* herb

**Figure 3:**
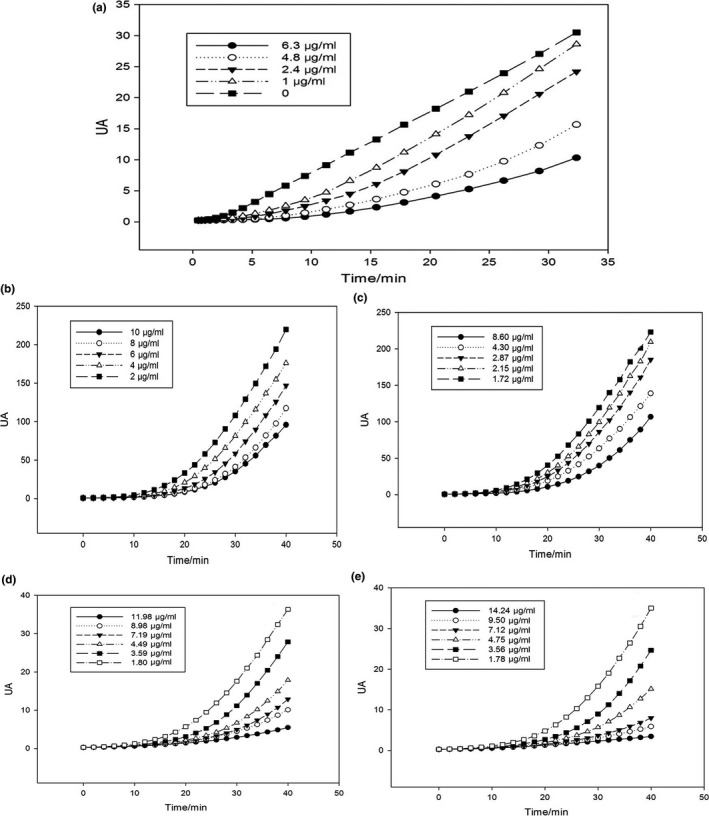
PSC determination curve of (a) ascorbic acid, (b) free phenols of bulbs, (c) free phenols of leaves, (d) free phenols of flowers, and (e) Bound phenols of flowers

**Figure 4:**
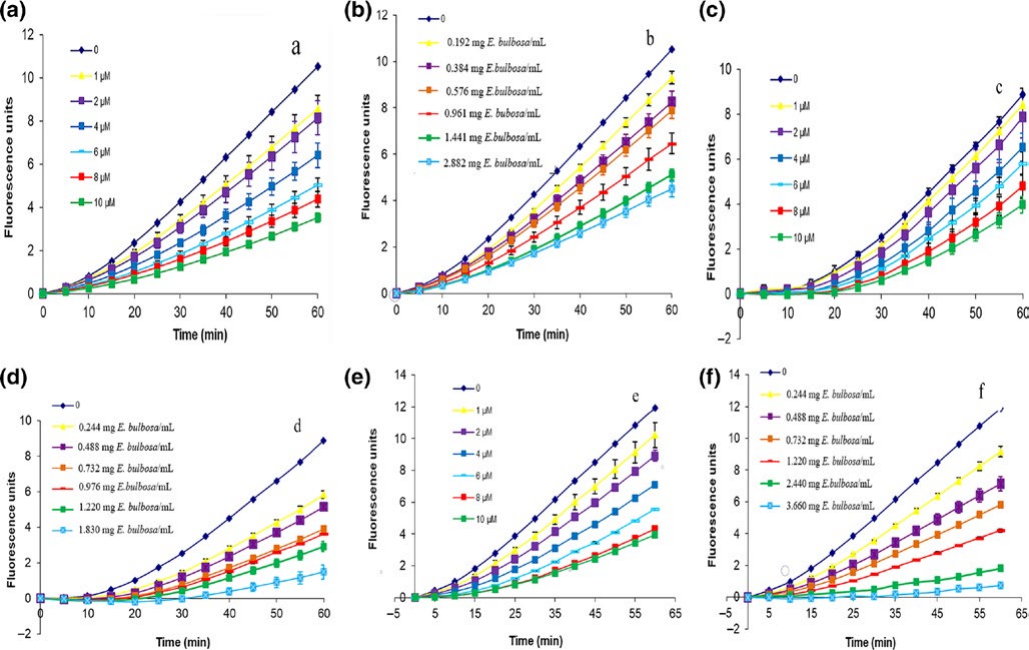
Time kinetics and dose‐response curve of (a) quercetin, (b) *Eleutherine bulbosa* bulbs, (c) quercetin, (d) *E. bulbosa* leaves, (e) quercetin, and (f) *E. bulbosa* flowers

PSC determination curves of free phenols of bulbs, leaves, flowers, and bound phenols of flowers were shown in Figure 3. The free phenols had a certain degree of oxidation resistance in a dose‐dependent manner. The phenols of stems and leaves were low in concentration which was beyond the range of determination, and flowers had the highest PSC value shown in Table 1. After integrating the curves of PSC, the Calcusyn software was used to calculate the PSC value. Antioxidant activity of bulbs, leaves, and flowers was related to the content of total phenols and flavonoids. The higher content of flavonoids or total phenols, the stronger the antioxidant capacity. The kinetics of DCFH oxidation in HepG2 cells generated from ABAP was shown in Figure 4. The increase in fluorescence intensity from DCF formation was inhibited by extracts in a dose‐dependent manner, as demonstrated by the curves generated from cells treated with quercetin and extraction of propolis alcohol. Quercetin and propolis alcohol solution could significantly reduce the fluorescence intensity, which indicated that extraction of propolis alcohol reduced radical peroxyl levels. This showed that the phenol had antioxidant effect, and the antioxidant effect increased with the increase in extract concentration. The parameter EC_50_ was defined as the required dose to cause 50% inhibition (PSC = 0.5) for each standard and sample extract. Antioxidant activity of the sample extract was expressed as quercetin equivalent 100 g dry weights. The EC_50_ values of quercetin and sample were shown in Table 2 along with equivalent quercetin content. Bulbs of *E. plicata* herb had the lowest EC_50_ value of 0.335 ± 0.018 mg/ml and the highest EQCvalue of 1198.21 ± 64.38 μM/100 g which indicated that bulbs had strongest anti‐peroxyl radical oxidation, followed by leaves and flowers.

**Table 1:** Total antioxidant activity of free phenols, bound phenols, and total phenols content in bulbs, leaves, and flowers of *Eleutherine bulbosa* (NTa means the PSC value could not be tested)

	Total antioxidant activity (mg VCE per 100 g DW)		
	Free	Bound	Total
*E. bulbosa* bulbs	611.11 ± 23.22	NTa	611.11 ± 23.22
*E. bulbosa* leaves	528.77 ± 19.83	NTa	528.77 ± 19.83
*E. bulbosa* flowers	2,093.60 ± 108.89	20.49 ± 0.36	2114.09 ± 109.12

**Table 2:** EC_50_ values for the inhibition of peroxyl radical and equivalent quercetin content (EQC)

	Control group	Sample	Group
	EC_50_ (μM)	EC_50_ (μM)	EQC (μM/100 g)
*Eleutherine bulbosa* flowers	5.03 ± 0.31	1.48 ± 0.08	339.86 ± 17.09
*E. bulbosa* leaves	5.41 ± 0.44	0.645 ± 0.056	838.76 ± 72.82
*E. bulbosa* bulbs	4.014 ± 0.234	0.335 ± 0.018	1198.21 ± 64.38

## 4 CONCLUSION

In this study, the contents of phenols and flavonoids in different parts of *E. bulbosa* were detected. The content of total phenols and flavonoids was in the order of flowers > bulbs > leaves. Antioxidant capacity test showed that the ascorbic acid equivalent of flowers is much higher than the content of stems and leaves which means that the order of antioxidant capacity in vitro is flowers > bulbs > leaves. Antioxidant capacity was positively correlated with flavonoids or total phenols content. The higher content of flavonoids or total phenols, the stronger the antioxidant capacity. The results presented that the order of the antioxidant activity of the extract in vitro was bulbs > leaves > flowers. The different results of the antioxidative activity may be due to the fact that some substances have a high antioxidant capacity and may have almost no antioxidant activity in the body. The antioxidant activity of *E. bulbosa* extracts showed it's certain nutritional value and therefore had the potential as a source of natural antioxidants.

## CONFLICT OF INTEREST

There are no conflict of interests.

## ETHICAL STATEMENTS

The current study was not required to complete an ethical assessment.
